# Early Loss of Blood-Brain Barrier Integrity Precedes *NOX2* Elevation in the Prefrontal Cortex of an Animal Model of Psychosis

**DOI:** 10.1007/s12035-016-9791-8

**Published:** 2016-02-24

**Authors:** Stefania Schiavone, Emanuela Mhillaj, Margherita Neri, Maria Grazia Morgese, Paolo Tucci, Maria Bove, Mario Valentino, Giuseppe Di Giovanni, Cristoforo Pomara, Emanuela Turillazzi, Luigia Trabace, Vincenzo Cuomo

**Affiliations:** 1 Department of Clinical and Experimental Medicine, University of Foggia, Foggia, Italy; 2 Department of Physiology and Pharmacology, “Sapienza”, University of Rome, Rome, Italy; 3 Department of Physiology and Biochemistry, University of Malta, Msida, Malta

**Keywords:** Blood-brain barrier, Oxidative stress, Social isolation, Psychosis, NADPH oxidase

## Abstract

The social isolation rearing of young adult rats is a model of psychosocial stress and provides a nonpharmacological tool to study alterations reminiscent of symptoms seen in psychosis. We have previously demonstrated that social isolation in rats leads to increased oxidative stress and to cerebral NOX2 elevations. Here, we investigated early alterations in mRNA expression leading to increased NOX2 in the brain. Rats were exposed to a short period of social isolation (1 week) and real-time polymerase chain reaction (PCR) for mRNA expression of genes involved in blood-brain barrier (BBB) formation and integrity (*ORLs*, *Vof 21* and *Vof 16*, *Leng8*, *Vnr1*, and *Trank 1 genes*) was performed. Real-time PCR experiments, immunohistochemistry, and Western blotting analysis showed an increased expression of these genes and related proteins in isolated rats with respect to control animals. The expression of specific markers of BBB integrity, such as matrix metalloproteinase 2 (MMP2), matrix metalloproteinase 9 (MMP9), occludin 1, and plasmalemmal vesicle associated protein-1 (PV-1), was also significantly altered after 1 week of social isolation. BBB permeability, evaluated by quantification of Evans blue dye extravasation, as well as interstitial fluid, was significantly increased in rats isolated for 1 week with respect to controls. Isolation-induced BBB disruption was also accompanied by a significant increase of Interleukin 6 (IL-6) expression. Conversely, no differences in NOX2 levels were detected at this time point. Our study demonstrates that BBB disruption precedes NOX2 elevations in the brain. These results provide new insights in the interplay of mechanisms linking psychosocial stress to early oxidative stress in the brain, disruption of the BBB, and the development of mental disorders.

## Introduction

Recent experimental evidence reported that psychosocial stress induces an increased production of reactive oxygen species (ROS) in the central nervous system (CNS) and that prolonged oxidative injury contributes to the development of mental disorders, in particular, psychosis [1, 2]. The nicotinamide adenine dinucleotide phosphate (NADPH) oxidases (NOX) are proteins that transfer electrons across biological membranes and are a source of superoxide. This family includes seven members (i.e., NOX1-5 and DUOX1-2), with distinct tissue distribution and mechanisms of activation [3]. In the CNS, the NADPH oxidase 2 enzyme (NOX2) is involved in cell fate and modulation of neuronal activity [4]. From a pathological point of view, NOX-derived increase of oxidative stress is thought to play a crucial role in several brain disorders, such as neurodegenerative diseases and psychiatric disorders [1, 5]. In a rodent model of psychosis obtained by the administration of subanaesthetic dose of the N-methyl-d-aspartate (NMDA)-receptor antagonist ketamine, NOX2 has been shown to be a major producer of ROS in the brain, controlling glutamate release and, ultimately, leading to behavioral alterations [6, 7]. Also, the decrease of parvalbumin GABAergic neurons observed in this animal model was prevented by NOX2 deficiency and by the treatment with the antioxidant/NOX inhibitor apocynin [7, 8]. The social isolation rearing of young adult rats is defined as a model of psychosocial stress [9, 10] and provides a nonpharmacological tool to study long-term alterations reminiscent of several symptoms seen in psychotic patients [11–13], as well as the decrease in parvalbumin-positive neurons [14]. We previously demonstrated that NOX2 contributes to the development of behavioral and neuropathological alterations in isolated rats [15] and that social isolation induces rapid elevations of the NOX2 complex in specific brain areas, such as nucleus accumbens and prefrontal cortex [1]. However, primary neuropathological events leading to early NOX2 elevations in the brain have not yet been established. Accumulating evidence from rodents, as well as human studies, showed a key contribution of blood-brain barrier (BBB) dysfunction, as well as BBB disruption-induced neuroinflammatory processes [16, 17], in the pathogenesis of psychiatric illnesses [18–20].

Here, we investigated the possible link between psychosocial stress-induced early NOX2 elevations in the prefrontal cortex and BBB disruption. Using molecular biology tools in combination with immunohistochemistry, we demonstrate that the loss in BBB integrity precedes the observed elevations in cerebral NOX2, possibly being its leading cause.

## Materials and Methods

### Animals

An equal number of adult male and female Wistar rats (Harlan, S. Pietro al Natisone, Udine, Italy) were used to obtain litters. All animals were housed at a constant room temperature (22 ± 3 °C) and relative humidity (55 ± 5 %) under a 12-h light/dark cycle (lights on from 7:00 AM to 7:00 PM). Food and water were freely available. Procedures involving animals and their care were conducted in conformity with the institutional guidelines of the Italian Ministry of Health (D.L. 26/2014), the Guide for the Care and Use of Mammals in Neuroscience and Behavioral Research (National Research Council 2004), the European Parliament directive 2010/63/EU and the Council of 22 September 2010 on the protection and use of animals for scientific purposes. All procedures involving animals were conducted in accordance with the ARRIVE guidelines. Animal well-being was daily monitored over the entire period of the experiments conducted. No signs of distress were evident, and all efforts were made to minimize the number of animals and their suffering.

### Social Isolation Protocol

The social isolation procedure was performed on male rats following the protocol described by Leng et al. [10]. At weaning (postnatal day 21), male pups were separated from their mothers and reared either in isolation (ISO: one rat per cage) or in social groups (GRP: four or five rats per cage). To avoid a litter effect, one subject was put in the control group and the other reared in isolation. All animals were reared in Plexiglas cages (48.0 × 27.0 × 20.0 cm for the isolated; 59.0 × 38.5 × 20.0 cm for the controls) and disturbed only for cleaning purposes. Isolated and control rats were housed in the same room so that isolated rats maintained visual, auditory, and olfactory contact with controls. The postweaning social isolation was performed for a period of 1 week.

### Blindness of the Study

Histological and biomolecular analyses were performed blind with respect to the rearing conditions. The blinding of the data was maintained until the analysis was terminated.

### RNA Extraction

Rats were decapitated under anaesthesia, and the brain was immediately removed for dissection. Tissues were frozen and stored at −80 ° C until the analysis was performed. Total RNA was extracted from the prefrontal cortex of control and isolated rats with TRIZOL Reagent (GibcoBRL, Rockville, MD) according to the manufacturers’ protocol. The total RNA was then purified and quantified as previously described [6, 15].

### cDNA Synthesis

Complementary DNA (cDNA) was synthesized using the ABI High Capacity cDNA kit (Applied Biosystems, Life Technologies, Paisley, UK), as previously described [6, 15]. The reverse transcription reaction was performed in a thermocycler (PTC-200, MJ Research) with a three-step program: 10 min at 25 °C followed by 120 min at 37 °C and a final 5 min step at 85 °C. The machine was set to cool down automatically to 4 °C after the end of the final step. cDNA samples were then used immediately for real-time polymerase chain reaction (PCR) or frozen and stored at −20 °C until needed.

### Real-Time Quantitative PCR

Real-time quantitative PCRs were performed, as previously described [6]. The target genes ORLs (ID: 29256), Vof21 (ID: 259228), Vof16 (ID: 259227), Leng8 (ID: 361506), Vnr1 (ID: 286893), Trank1 (ID: 316022), and the housekeeping gene of reference GAPDH (ID: 24383) were identified by FAM-labeled probes.

### Reverse Transcriptase PCR

Reverse transcriptase PCRs were performed as previously described [15]. Briefly, they were performed at 35 cycles for target genes and at 30 cycles for the housekeeping gene-actin. PCR conditions, primers, and product size have been previously described [21].

### Immunohistochemistry

Immunohistsochemical analyses were performed as previously described [15]. Brain sections were deparaffinized through graded alcohols, subjected to heat-induced epitope retrieval for 15 min in 0.01 mol/L citrate buffer (pH 6.0) and incubated for 2 h at room temperature in PBS-albumin blocking serum buffer containing antibodies raised against oxidized low-density lipoprotein (lectine-like) receptors (ORLs) (1:100, Novusbio), leucocyte receptor cluster member 8 (Leng8) (1:200 Novusbio), vomoronasal receptor-1 (Vnr1) (1:300 Abcam), and tetratricopeptide repeat and ankyrin repeat containing1 (Trank1) (1:100 Novusbio). Sections were then incubated for 15 min at room temperature with specific biotinylated secondary antibody and, after several washes in PBS, for 15 min in horseradish peroxidase-avidin/biotin complex solution. Horseradish peroxidase was visualized using 3,3-diaminobenzidinetetrahydrochloride hydrate (DAB, Sigma-Aldrich) and H_2_O_2_. Counterstaining with hematoxylin-eosin allowed visualization of cell morphology and nuclei by light microscopy.

### Confocal Microscopy

Confocal microscopy analysis was performed as previously described [22], using a True Confocal Scanner, Leica TCS SPE confocal microscope with a final three-dimensional image reconstruction.

### Western Blotting

Western blot analysis was performed according to the standard procedure, using the following antibodies: oxidized low-density lipoprotein (lectine-like) receptors (ORLs) (1:1000, Novusbio), leucocyte receptor cluster member 8 (Leng8) (1:2000 Novusbio), vomoronasal receptor-1 (Vnr1) (1:2000 Abcam), tetratricopeptide repeat and ankyrin repeat containing1 (Trank1) (1:1000 Novusbio), matrix metalloproteinase 9 (MMP9) (1:1000 Abcam), matrix metalloproteinase 2 (MMP2) (1:1000 Abcam), occludin 1 (1:1000, Abcam), plasmalemmal vesicle associated protein-1 (PV-1) (1:2000 Abcam), IL-6 (1:2000 Santa Cruz, CA, USA), anti-TNF (1:600 Santa Cruz, CA, USA), anti-IL-1 beta (1:4000 Santa Cruz, CA, USA), anti-IL-10 (1:2000 Peprotec, London, UK), and beta-actin (1:4000, Sigma-Aldrich). Fifty micrograms of proteins per lane were diluted in loading buffer and denatured at 70 °C for 10 min. Optical densities of the bands were measured using ImageJ software (http://rsb.info.nih.gov/ij/) and normalized against beta-actin.

### Evaluation of BBB Integrity

#### Quantification of Evans Blue Dye Extravasation

Quantification of Evans blue dye extravasation was performed as previously described [23, 24]. Briefly, rats were intracardially perfused with warm 0.9 % NaCl to wash out blood cells, followed by 0.5 % Evans blue in cold 4 % PFA. After perfusion, the brain was taken out, weighed, and homogenized in 50 % trichloroacetic acid solution. Brain samples were then centrifuged at 10,000×*g* for 10 min at 4 °C. Supernatants were then measured by spectrophotometer (Perkinelmer, Italy) at 620 nm. The total Evans blue content (ng) was calculated according to the external standard curve and expressed as nanograms of Evans blue dye per milligram of tissue. Evans blue assessment of BBB integrity was performed on the whole brain (right and left hemispheres). In Fig. 5a, a representative image of the left hemisphere was shown, given that the extravasation of the blue dye was particularly present in the internal (interhemispheric) sides of the two hemispheres. No significant differences in the amount of the presence of extravasated dye between right and left hemispheres were detected (data not shown).

#### Quantification of Brain Interstitial Fluid

Quantification of brain interstitial fluid (IF) was performed as previously described [25]. Briefly, after rats’ sacrifice, brains were removed and the wet weight was weighed out. The brains were then dried overnight to a constant weight an oven at 110 °C. The % of brain IF was calculated according to the following formula:

$$ \%\ \mathsf{IF}=\left[\left(\mathsf{Wet}\kern0.5em \mathsf{weight}\hbox{-} \mathsf{Dry}\kern0.5em \mathsf{weight}\right)/\mathsf{Wet}\kern0.5em \mathsf{weight}\right]\mathsf{x}\mathsf{100} $$

### Statistical Analysis

Data were analyzed using GraphPad Prism 5 software for Windows. Data were analyzed by unpaired Student’s *t* test. For all tests, a *P* < 0.05 was considered statistically significant. Results are expressed as means ± mean standard error (SEM).

## Results

### mRNA Expression of Genes Related to BBB Disruption and Neuroinflammation Is Increased in the Prefrontal Cortex of Rats After 1 Week of Social Isolation

We analyzed if social isolation might induce early alterations in the mRNA expression of genes involved in BBB formation and maintenance of its integrity. Moreover, we investigated if these alterations might occur before NOX2 elevations in the brain. To these purposes, rats were exposed to a period of 1 week of social isolation and real-time PCR of the prefrontal cortex was then performed.

In particular, we checked mRNA expression alterations of the following genes, which have been shown to be directly involved in BBB formation and integrity:

1. *ORLs genes:* they encode for receptors binding low-density lipoproteins resulting from vascular inflammatory processes, acting in association with NF-kappaB through increased ROS production and reduction of nitric oxide release (www.genecards.org; http://biit.cs.ut.ee/gprofiler). Elevations in the expression of these receptors have been considered as a valid biomarker of disruption of blood vessels formation, BBB inflammatory process, and increased permeability of this system [26, 27];
2. *Vof 21 and Vof 16 genes:* expression of these genes is thought to be increased after different cerebral insults, CNS vasculature inflammation, and disrupted formation of BBB tight junctions (www.genecards.org; http://biit.cs.ut.ee/gprofiler) [28];
3. *Leng 8*: this gene encodes for a receptor with a key role during the early phase of blood vessel morphogenesis. Increased expression and activation of this receptor have been observed in inflammatory processes of the vascular system, associated with increased BBB permeability and morphological alterations of CNS vasculature (www.genecards.org; http://biit.cs.ut.ee/gprofiler) [29];
4. *Vnr1* is a gene implicated in blood vessel formation and in the regulation of blood pressure both in periphery and in the CNS. Increased expressed levels have been reported in blood vessel disruption and increased permeability of the BBB following neuroinflammation (www.genecards.org; http://biit.cs.ut.ee/gprofiler) [30];
5. *Trank1* gene has been found to be increased in different pathological conditions associated to neuroinflammation, disrupted formation and functioning of blood vessels of the BBB (www.genecards.org; http://biit.cs.ut.ee/gprofiler).

**Fig. 1:**
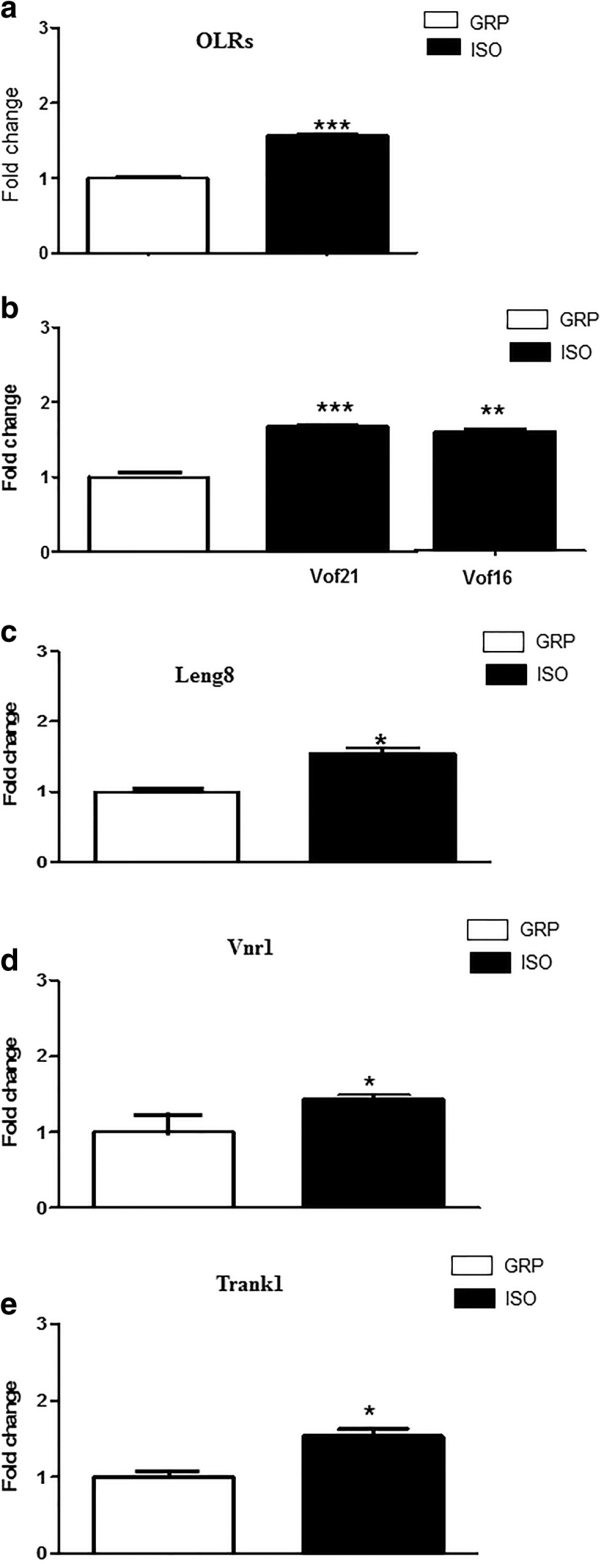
Increase of ORLs, Vof21, Vof16, Leng8, Vnr1, and Trank1 mRNA after 1 week of social isolation. **a** Real-time PCR quantification of ORLs mRNA in the prefrontal cortex of control (GRP) and 1-week isolated rats (ISO) (fold change of increase). ***P < 0.001, unpaired Student’s *t* test; *n* = 5 per group. **b** Real-time PCR quantification of Vof21 and Vof16 mRNA in the prefrontal cortex of control (GRP) and 1-week isolated rats (ISO) (fold change of increase). ***P < 0.001, **P < 0.01, unpaired Student’s t test; *n* = 5 per group. **c** Real-time PCR quantification of Leng8 mRNA in the prefrontal cortex of control (GRP) and 1-week isolated rats (ISO) (fold change of increase). *P < 0.05, unpaired Student’s *t* test; *n* = 5 per group. **d** Real-time PCR quantification of Vnr1 mRNA in the prefrontal cortex of control (GRP) and 1-week isolated rats (ISO) (fold change of increase). *P < 0.05, unpaired Student’s *t* test; *n* = 5 per group. **e** Real-time PCR quantification of Trank1 mRNA in the prefrontal cortex of control (GRP) and 1-week isolated rats (ISO) (fold change of increase). *P < 0.05, unpaired Student’s *t* test; *n* = 5 per group

Real-time PCR results revealed a statistically significant increase of the mRNA level of these genes in 1-week isolated rats with respect to control animals (Fig. 1a–e) (*n* = 5 per group; unpaired Student’s *t* test; ***P < 0.001 for ORLs and Vof 21; **P < 0.01 for Vof 16; *P < 0.05 for Leng8, Vnr1, and Trank1).

#### Immunostaining and Expression of Proteins Related to BBB Disruption and Neuroinflammation Are Increased in the Prefrontal Cortex of Rats After 1 Week of Social Isolation

**Fig. 2:**
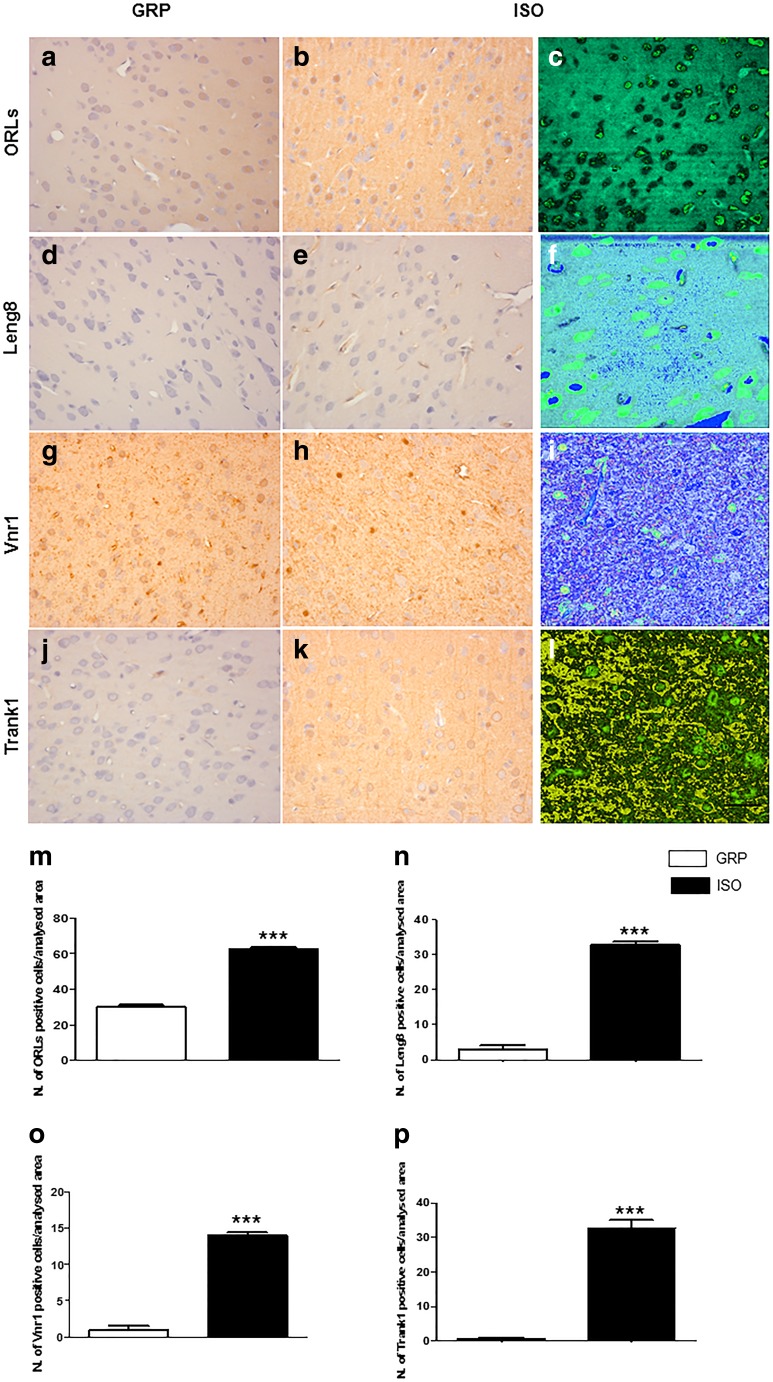
Increase of ORLs, Leng8, Vnr1, and Trank1 positive-stained cells after 1 week of social isolation. **a**, **b** Representative images of DAB immunohistochemistry staining for ORLs in the prefrontal cortex of control (**a**) and isolated (**b**) rats; *n* = 5 per group. **c** Representative image of confocal microscopy for ORLs in the prefrontal cortex of isolated animals; *n* = 5 per group. **d**, **e** Representative images of DAB immunohistochemistry staining for Leng8 in the prefrontal cortex of control (**d**) and isolated (**e**) rats; *n* = 5 per group. **f** Representative image of confocal microscopy for Leng8 in the prefrontal cortex of isolated animals; *n* = 5 per group**. g**, **h** Representative images of DAB immunohistochemistry staining for Vnr1 in the prefrontal cortex of control (**g**) and isolated (**h**) rats; *n* = 5 per group. i Representative image of confocal microscopy for Vnr1 in the prefrontal cortex of isolated animals; *n* = 5 per group. **j**, **k** Representative images of DAB immunohistochemistry staining for Trank1 in the prefrontal cortex of control (**j**) and isolated (**k**) rats; *n* = 5 per group. **l** Representative image of confocal microscopy for Trank1 in the prefrontal cortex of isolated animals; *n* = 5 per group. **m** Quantification of ORLs-immunoreactive cells in the prefrontal cortex of control (GRP) and 1-week isolated rats (ISO). ***P < 0.001, unpaired Student’s *t* test; *n* = 5 per group. **n** Quantification of Leng8-immunoreactive cells in the prefrontal cortex of control (GRP) and 1-week isolated rats (ISO). ***P < 0.001, unpaired Student’s *t* test; *n* = 5 per group. **o** Quantification of Vnr1-immunoreactive cells in the prefrontal cortex of control (GRP) and 1-week isolated rats (ISO). ***P < 0.001, unpaired Student’s *t* test; *n* = 5 per group. **p** Quantification of Trank1-immunoreactive cells in the prefrontal cortex of control (GRP) and 1-week isolated rats (ISO). ***P < 0.001, unpaired Student’s *t* test; *n* = 5 per group

We investigated whether 1 week of social isolation was able to induce immunohistochemical alterations for ORLs, Leng8, Vnr1, and Trank1. Immunohistochemistry revealed a significant difference in the number of ORLs positive stained cells between controls and 1-week isolated animals (Fig. 2a, b, m) (***P < 0.001, unpaired Student’s *t* test; *n* = 5 per group). A significant elevation in the number of Leng8 positive cells was also detected in isolated rats with respect to animals reared in group (Fig. 2d–e, n) (***P < 0.001, unpaired Student’s *t* test; *n* = 5 per group). While in control animals there were virtually no Vnr1 positive cells, a strong increase of Vnr1 staining was detected after 1 week of social isolation (Fig. 2g–h, o) (***P < 0.001, unpaired Student’s *t* test; *n* = 5 per group). This isolation period also led to a significant elevation of Trank1 positive stained cells with respect to nonisolated animals, where virtually no staining for Trank 1 was detected (Fig. 2j **–**k, p) (***P < 0.001, unpaired Student’s *t* test; *n* = 5 per group). Confocal microscopy analysis revealed the intracellular and/or transmembrane distribution of these protein immunoreactivity (Fig. 2c, f, i, l).

**Fig. 3:**
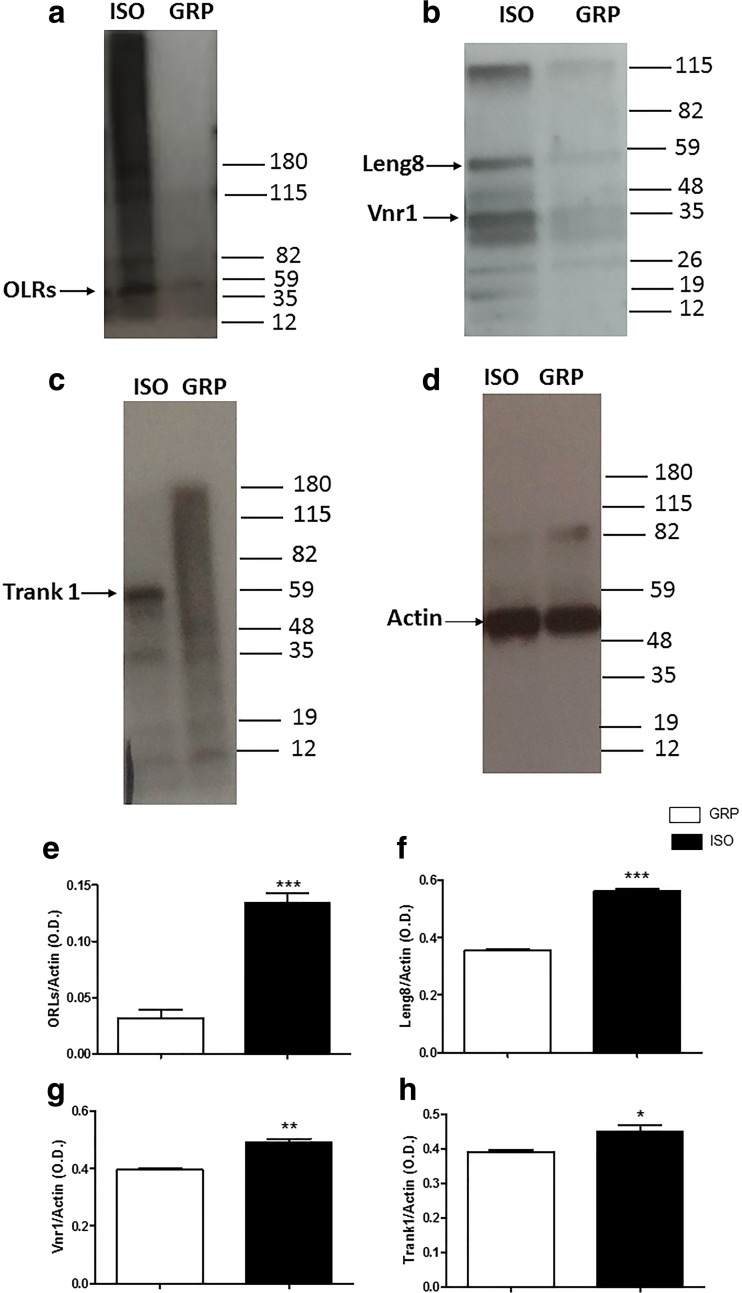
Increase of ORLs, Leng8, Vnr1, and Trank1 protein levels after 1 week of social isolation. **a** Representative images of western blotting for ORLs in the prefrontal cortex of control (GRP) and 1-week isolated rats (ISO), *n* = 3 per group. **b** Representative images of western blotting for Leng8 and Vnr1 in the prefrontal cortex of control (GRP) and 1-week isolated rats (ISO), *n* = 3 per group. **c** Representative images of western blotting for Trank 1 in the prefrontal cortex of control (GRP) and 1-week isolated rats (ISO), *n* = 3 per group. **d** Representative images of Western blotting for actin in the prefrontal cortex of control (GRP) and 1 week isolated rats (ISO), *n* = 3 per group. **e** Quantification of the optical density of ORLs band normalized to the actin protein value in the prefrontal cortex of control (GRP) and 1-week isolated rats (ISO), ***P < 0.001, unpaired Student’s *t* test; *n* = 3 per group. **f** Quantification of the optical density of Leng8 band normalized to the actin protein value in the prefrontal cortex of control (GRP) and 1-week isolated rats (ISO), ***P < 0.001, unpaired Student’s *t* test; *n* = 3 per group. **g** Quantification of the optical density of Vnr1 band normalized to the actin protein value in the prefrontal cortex of control (GRP) and 1-week isolated rats (ISO), **P < 0.01, unpaired Student’s *t* test; *n* = 3 per group. **h** Quantification of the optical density of Trank1 band normalized to the actin protein value in the prefrontal cortex of control (GRP) and 1-week isolated rats (ISO), *P < 0.05, unpaired Student’s *t* test; *n* = 3 per group

To analyze if increased expression of mRNA levels of the genes in isolated animals was associated to elevation in protein expression, we performed Western blot analysis for ORLs, Leng8, Vnr1, and Trank1. A significant increase in the expression of ORLs (Fig. 3a, e) (***P < 0.001, unpaired Student’s *t* test; *n* = 3 per group), Leng8 (Fig. 3b, f) (***P < 0.001, unpaired Student’s *t* test; *n* = 3 per group), Vnr1 (Fig. 3b, g) (**P < 0.01, unpaired Student’s *t* test; *n* = 3 per group), and Trank1 (Fig. 3c, h) (*P < 0.05, unpaired Student’s *t* test; *n* = 3 per group) protein levels was detected in 1-week isolated animals as compared to control rats.

#### Expression of Markers of BBB Loss of Integrity Is Altered After 1 Week of Social Isolation

**Fig. 4:**
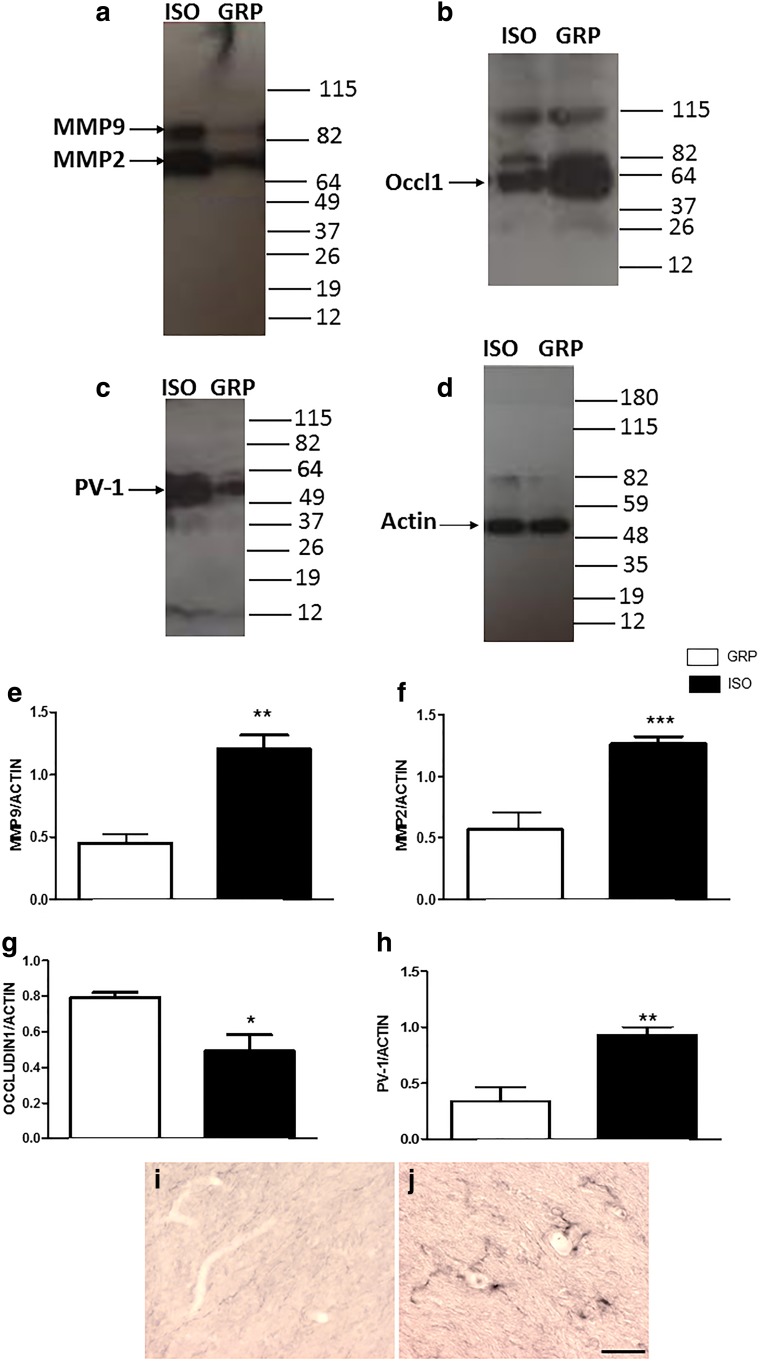
Alterations of MMP9, MMP2, occludin 1, and PV-1 proteins after 1 week of social isolation. **a** Representative images of Western blotting for MMP9 and MMP2 in the prefrontal cortex of control (GRP) and 1-week isolated rats (ISO), *n* = 5 per group. **b** Representative images of Western blotting for occludin 1 in the prefrontal cortex of control (GRP) and 1-week isolated rats (ISO), *n* = 5 per group. **c** Representative images of Western blotting for PV-1 in the prefrontal cortex of control (GRP) and 1-week isolated rats (ISO), *n* = 5 per group. **d** Representative images of Western blotting for actin in the prefrontal cortex of control (GRP) and 1-week isolated rats (ISO), *n* = 5 per group. **e** Quantification of the optical density of MMP9 band normalized to the actin protein value in the prefrontal cortex of control (GRP) and 1-week isolated rats (ISO), **P < 0.01, unpaired Student’s *t* test; *n* = 5 per group. **f** Quantification of the optical density of MMP2 band normalized to the actin protein value in the prefrontal cortex of control (GRP) and 1-week isolated rats (ISO), ***P < 0.001, unpaired Student’s *t* test; *n* = 5 per group. **g** Quantification of the optical density of occludin 1 band normalized to the actin protein value in the prefrontal cortex of control (GRP) and 1-week isolated rats (ISO), *P < 0.05, unpaired Student’s *t* test; *n* = 5 per group. **h** Quantification of the optical density of PV-1 band normalized to the actin protein value in the prefrontal cortex of control (GRP) and 1-week isolated rats (ISO), **P < 0.01, unpaired Student’s *t* test; *n* = 5 per group. **i**, **j** Representative images of DAB immunohistochemistry staining for PV-1 in the prefrontal cortex of control (**i**) and isolated (**j**) rats; *n* = 5 per group

In order to investigate whether BBB integrity was affected by 1 week of social isolation, we performed Western blot analysis for specific markers of BBB disruption that included MMP9, MMP2, occludin 1 [31], and PV-1 [32]. Results obtained through Western blotting showed that 1 week of social isolation induced a significant increase of MMP9 (Fig. 4a, e) (**P < 0.01, unpaired Student’s *t* test; *n* = 5 per group) and MMP2 (Fig. 4a, f) (***P < 0.001, unpaired Student’s *t* test; *n* = 5 per group) as compared to nonisolated rats. A significant increase of PV-1 protein levels in 1-week isolated animals was also shown by Western blot analysis (Fig. 4c, h) (**P < 0.01, unpaired Student’s *t* test; *n* = 5 per group), as well as by immunohistochemistry, which revealed a statistically significant increase of PV-1 staining after 1 week of social isolation in comparison to control animals (Fig. 4i, j). Conversely, expression levels of occludin 1 protein were significantly lower in isolated rats with respect to controls (Fig. 4b, g) (*P < 0.05, unpaired Student’s *t* test; *n* = 5 per group).

#### Loss of BBB Integrity After 1 Week of Social Isolation

**Fig. 5:**
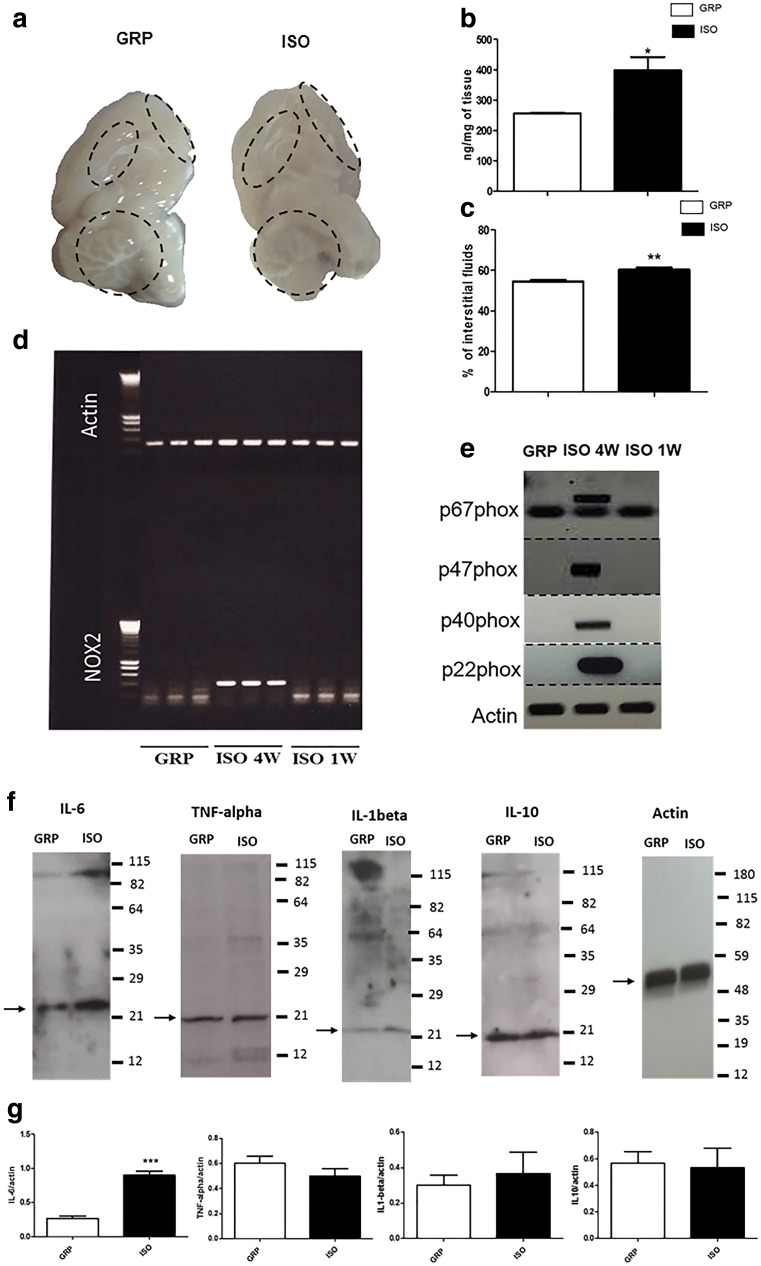
Increase of BBB permeability and IL-6 but not of NOX2 and its functional subunits after 1 week of social isolation. **a** Representative image of the left hemispheres of control (GRP) and 1-week isolated rats (ISO) after the Evans blue essay, *n* = 3 per group. **b** Quantification of extravasated dye (ng/mg of tissue) in the brain of control (GRP) and 1-week isolated rats (ISO) after the Evans blue essay. *P < 0.05, unpaired Student’s *t* test; *n* = 3 per group. **c** Quantification of interstitial fluid (%) in the brain of control (GRP) and 1-week isolated (ISO) rats. **P < 0.01, unpaired Student’s *t* test; *n* = 3 per group. **d** RT-PCR for actin and NOX2 mRNA in the prefrontal cortex of control (GRP), 4-week (ISO 4 W) and 1-week isolated rats (ISO 1 W), *n* = 5 per group. **e**) Representative image of RT-PCR for p67phox, p47phox, p40phox, p22phox and actin mRNA in the prefrontal cortex of control (GRP), 4 week (ISO 4W), and 1 week isolated rats (ISO 1W), *n* = 3 per group. **f** Representative image of Western blotting for IL-6, TNF-alpha, IL-1beta, IL-10, and actin in the prefrontal cortex of control (GRP) and 1-week isolated rats (ISO), *n* = 3 per group. **g** Quantification of the optical density of IL-6, TNF-alpha, IL-1beta, IL-10 band normalized to the actin protein value in the prefrontal cortex of control (GRP), and 1-week isolated rats (ISO). IL-6: ***P < 0.001, unpaired Student’s *t* test; *n* = 3 per group; TNF-alpha: *P* = 0.2879, unpaired Student’s *t* test; *n* = 3; IL-1beta: *P* = 0.6433, unpaired Student’s *t* test; *n* = 3 per group; IL-10 protein expression: *P* = 0.8541, unpaired Student’s *t* test; *n* = 3 per group

In order to investigate if 1 week of social isolation might cause increased permeability of the BBB, we performed Evans blue dye assay in control and isolated animals. Macroscopic analysis of brain hemispheres revealed the presence of dye extravasation in rats exposed to 1 week of social isolation with respect to controls (Fig. 5a). Importantly, there were no macroscopic signs of cerebral hemorrhage in isolated animals (Fig. 5a). Quantification of extravasated Evans blue dye revealed that 1 week of isolation led to a significant increased blue dye content with respect to the one found in the brain parenchyma of control animals (Fig. 5b) (*P < 0.05, unpaired Student’s *t* test; *n* = 3 per group). This was also associated with a significant elevation in the percentage of edema that was detected in brain tissue of isolated animals in comparison to nonisolated rats (Fig. 5c) (**P < 0.01, unpaired Student’s *t* test; *n* = 3 per group). Reverse transcriptase PCR results showed that, at the same time point, expression of NOX2 mRNA, as well as of its functional enzymatic subunits p67phox, p47phox, p40phox, and p22phox, was below detection levels both in control and 1-week isolated animals (Fig. 5d, e). Conversely, as previously shown, mRNA of NOX2 was significantly increased after 4 weeks of social isolation with respect to nonisolated animals [1]. In order to further confirm this observation and to obtain a comparative positive control for the reverse transcriptase PCR conditions used in the present manuscript, we included, for this experiment, three rats exposed to social isolation for a period of 4 weeks (Fig. 5d). The increase of NOX2 mRNA observed in these animals was also associated with an elevation in the expression of p67phox, p47phox, p40phox, and p22phox functional subunits (Fig. 5e). These three animals began the social isolation protocol at the same time point and under the same experimental conditions to those experimental animals that remained isolated for 1 week.

#### Increase of IL-6 Protein Expression Is Associated to Isolation-Induced BBB Disruption

In order to verify if 1-week social isolation-induced BBB disruption was associated to neuroinflammatory processes which might induce the cortical increased NOX2 expression previously observed at later time points [1], we analyzed IL-6, TNF-alpha, IL-1beta, and IL-10 protein levels by Western blotting. Social isolation-induced BBB disruption was associated to a specific increase of IL-6 with respect to control animals (***P < 0.001, unpaired Student’s *t* test; *n* = 3 per group), while no significant differences in TNF-alpha (*P* = 0.2879, n.s., unpaired Student’s *t* test; *n* = 3 per group) IL-1beta (*P* = 0.6433, n.s., unpaired Student’s *t* test; *n* = 3 per group) and IL-10 protein expression (*P* = 0.8541, n.s., unpaired Student’s *t* test; *n* = 3 per group) were detected between animals reared in isolation and in group for a period of 1 week (Fig. 5f).

## Discussion

In this study, we investigated early mRNA expression alterations leading to the increase of NOX2 in the prefrontal cortex of an animal model of psychosis, such as the social isolation rearing of rats. We demonstrated that a short period of social isolation (1 week) leads to increased expression of mRNA of specific genes (*ORLs*; *Vof 21* and *Vof16*, *Leng8*, *Vnr1*, and *Trank1*) that are typically involved in cerebral blood vessel morphogenesis, BBB formation and integrity, as well as neuroinflammation. We hypothesized that increased expression of these mRNA genes might ultimately lead to an increase of NOX2, as previously reported in the rat prefrontal cortex after 4 weeks of social isolation [1], probably via increase in IL-6 expression.

Our results are in line with recent observations in humans and rodent models that report the crucial role of BBB disruption in the pathogenesis of mental disorders. Thus, dysfunctions of BBB have previously been shown in schizophrenic subjects [33], depressed patients with attempted suicide [34], as well as in patients suffering from bipolar disorders [35]. Moreover, psychiatric patients showed higher CSF/serum albumin ratio, a standard biomarker for altered BBB function and integrity as compared to controls [36]. Accordingly, damage to the BBB has also been reported in patients with neuropsychiatric syndromes (including delirium, depression, anxiety, and psychosis) and in systemic lupus erythematosus [37]. Long-term increase in BBB permeability and loss of integrity have also been demonstrated in individuals exposed to a number of neurotoxic compounds, such as heavy metals or drugs, acting with genetics and other brain damage mechanisms and resulting in an increased potential for the development of schizophrenia [38]. In a rodent model of psychosis-like posttraumatic syndrome, animals exhibited disruption of the BBB by virtue of increased BBB permeability as detected by horseradish peroxidase [39].

In our study, we observed an increased expression of mRNA of genes involved in BBB functioning after 1 week of postweaning social isolation, when animals could therefore be considered at a young age. In line with this aspect, Falcone and coworkers demonstrated that development of first psychotic episodes in youths was strongly associated with neuroinflammation and BBB dysfunction [40]. Also, young patients with first-onset schizophrenia showed increased cerebrospinal fluid and serum levels of S100B, a marker of failed BBB integrity and functioning impairment [41].

Previous studies in rodents have shown a role of acute stress (induced, for example, by restraint and immobilization) in increasing BBB permeability [42], mainly through activation and stimulation of mast cells or corticotropin-releasing hormone (CRH)-induced release of specific cell mediators [43]. However, to the best of our knowledge, no available data are present demonstrating a crucial role of early psychosocial stress in inducing BBB disruption.

We also demonstrated that 1 week of social isolation caused altered expression of specific proteins implicated in the maintenance of BBB integrity and functioning, i.e., MMP2, MMP9, occludin, and PV-1. Interestingly, these alterations are not concomitant to NOX2 elevations or its functional subunits p67phox, p47phox, p40phox, and p22phox in the prefrontal cortex of isolated animals. Indeed, we found no differences in NOX2 or its enzymatic subunits in 1-week isolated animals with respect to what was observed in animals reared in group. Hence, it could be hypothesized that loss of integrity and increased BBB permeability might serve as *primum movens* for NOX2 expression, activation and consequent production of ROS, as previously observed after 4 weeks of social isolation [1]. In support of this hypothesis and of an existence of a molecular link between altered expression of BBB disruption markers and ROS production, Haorah and colleagues demonstrated that MMP9 and MMP2 were directly activated by ROS via a specific tyrosine kinase, leading ultimately to degradation of the tight junctions of the BBB [44]. Accordingly, MMP inhibition by p-aminobenzoyl-gly-pro-D-leu-ala-hydoxamate has been shown to prevent oxidative stress-associated BBB disruption after transient focal cerebral ischemia [45]. Decreased expression of occludin has also been shown to be associated with increased BBB permeability in in vivo models of hypoxia/reoxygenation stress [46]. Additionally, pathological stressors seem to cause trafficking of occludin away from BBB tight junction protein complexes [47]. This occludin relocalization has been shown to be prevented in vivo by treatment with TEMPOL, an antioxidant that readily crosses the BBB [47].

In this study, we also showed that isolation-induced BBB disruption is associated to the presence of neuroinflammatory processes and, in particular, to an increase of IL-6 protein levels. In this context, a possible molecular and temporal link between early loss of BBB integrity and increased NOX2 expression observed at later time points [1] might be represented by neuroinflammation which is known to be induced by BBB disruption [16, 17]. In particular, activated microglia has been shown to modulate expression of tight junctions and, on the other hand, the endothelium can regulate the state of microglial activation [48]. However, we previously observed that nor microglia proliferation or activation were affected by 2 or 4 weeks of social isolation [1], suggesting that the increase of NOX2-derived oxidative stress is not mainly due to microglia proliferation and activation. Conversely, production of IL-6 has been shown to be necessary and sufficient for NOX2 increase in the ketamine model of schizophrenia [49], as well as the degeneration of forebrain GABAergic interneurons, along with cognitive impairment in aged mice, through activation of the neuronal NADPH oxidase [50]. Importantly, removal of IL-6 in neuronal cultures or in vivo, by using IL-6-deficient mice, prevented the superoxide increase induced by ketamine [49]. Undoubtedly, further investigations will be necessary to identify molecular pathways leading to IL-6 increase in the social isolation model.

To the best of our knowledge, this is the first in vivo report demonstrating a temporal relationship between BBB disruption (occurring as a very early event, after 1 week of social isolation) and increased expression of NOX2 and its functional enzymatic subunits. Thus, although in a study of Kahles et al., a role of NOX2 in mediating early BBB disruption in an animal model of experimental stroke has been demonstrated by the NOX2 knockout mice and apocynin treatment [51], no specific temporal relation between these two events was investigated. In a recent study, Rochfort and colleagues demonstrated that increased expression and coassociation of gp91phox and p47phox, two pivotal NADPH oxidase subunits, occurred in response to specific cytokine release induced by BBB disruption and that cytokine-dependent effects on ROS generation and endothelial permeability could be attenuated using antioxidant strategies (such as superoxide dismutase, catalase, N-acetylcysteine, apocynin) and targeting NADPH oxidase blockade [52]. However, this study was entirely conducted in vitro using primary-derived human brain microvascular endothelial cells.

Several recent studies, using animal models of neurological disorders induced by acute ischemic stroke, reported the presence of brain hemorrhage as a direct consequence of increased BBB permeability and disruption [53]. In contrast, in the animal model of psychosis used in the present study, loss of BBB integrity and increased permeability were not associated with any major signs of cerebral hemorrhage. Thus, it was possible to exclude any hemorrhage-induced neuropathological alterations in isolated animals. Therefore, the histological and biomolecular alterations, as previously reported to be a consequence of social isolation [1], cannot be attributed to a hemorrhagic event occurring at an early stage of social isolation rather than to NOX-derived increase of ROS production.

In the present study, we postulate that the increase in mRNA expression of genes can be considered as markers of BBB disruption and represents a very early event, which might also precede the appearance of psychotic-like symptoms in animals. We previously showed that a period of 2 weeks of social isolation did not induce any increase in locomotor activity, or a decrease in the discrimination index, which were conversely observed at later time points of social isolation period (4 and 7 weeks) [1]. In support to our observations, human studies point towards a role of BBB disruption and consequent cerebral inflammation in inducing psychiatric disorders occurring during the first episodes of psychosis in children [40].

It has been recently demonstrated that imprisonment involving greater levels of isolation and deprivation of social contacts were associated with higher rate of suicide and development of other psychiatric comorbidities [54]. In another recent study, isolation, such as the one applied in imprisonment for security reasons, has been identified as the major risk factor for the development of severe mental disorders and suicidal behavior [55]However, in these studies, no molecular pathways have been put forward to explain the link between human isolation and the consequent development of psychiatric disorders. Therefore, a possible role of increased oxidative stress or early BBB alterations could be a plausible explanation and should be verified in prison population.

In conclusion, our data support the existence of a pathological link between loss in BBB integrity, increased NOX-derived oxidative stress in the brain, and the development of psychosis-like neuropathological alterations. Results of the present study could open innovative insights in the identification of new biomarkers potentially useful for early or postmortem diagnosis of psychosis. Finally, the identification of novel mechanisms and signaling pathways could provide potential and promising innovative therapeutic approaches for the treatment of psychiatric disorders.
